# Distribution and Protective Role of HLAB40 in Iranian Patients with Kawasaki Disease; a Report from Southern Iran

**Published:** 2014-05-24

**Authors:** Gholamhossein Ajami, Khashayar Aflaki, Soheila Alyasin, Behrooz Gharesi-fard, Mohammad Borzouee, Hamid Amoozgar

**Affiliations:** 1 Department of Pediatrics Cardiology; 2 Department of Pediatrics Intensive Care; 3 Department of Pediatric Immunology; 4 Department of Immunology; 5 Cardiac and Neonatology Research Center, Shiraz University of Medical Sciences, Shiraz, Iran

**Keywords:** Kawasaki Disease, Gene Protection, Human Leukocyte Antigens, HLA- B40* Allele

## Abstract

***Objective:*** Kawasaki disease (KD) clinically presents as a systemic vasculitis syndrome with significant cardiovascular involvement. With different incidence among different ethnic groups, the role of certain human leukocyte antigens and their products has been considered as a crucial predisposing factor in the immune responses in this disease.

***Methods:*** We determined the distribution of human leukocyte antigens type B for 90 Iranian patients with Kawasaki disease in order to evaluate a possible association between these antigens and this disease in our area. We used the polymerase chain reaction (PCR) sequence specific primers (PCR-SSP) technique for antigen typing. Distribution of these antigens for 89 healthy Iranians used as control.

***Findings*** ***:*** While 7 (3.9%) of our patients were positive for human leukocyte antigen type B 40^*^, there were 18 (10.1%) subjects from the control group who had this antigen with statistically significant difference between patients and control group (CI= 95%, RR=1.15 and *P*= 0.02). Data were analyzed by Pearson chi-square test and Fisher's exact test. SPSS version 15 was used for statistical analysis and a *P* value less than 0.05 considered statistically significant

***Conclusion:*** The presence of higher frequency of allele type-B40^*^ in the control group may represent a protective role for this antigen with resultant decreased susceptibility to KD in our area.

## Introduction

Kawasaki disease (KD) is an acute febrile illness of childhood which presents as a systemic disease. While many organs are affected, involvement of cardiovascular system during early and late stages of the disease is considered as the leading cause of acquired heart disease in children in developed countries^[^^1^^,^^2^^]^. With more recognition of KD, it seems that the reported incidence is also increasing in some developing countries, even more than attacks of acute rheumatic fever in this age group^[^^3^^-^^5^^]^.

 While several decades have elapsed since Dr. Tomisaku Kawasaki described this disease for the first time, the cause is still unknown. Furthermore, epidemiological studies have revealed different incidence among different ethnic groups, and so the presence of certain human leukocyte antigens has been considered as a possible inherited predisposition for development of the disease^[^^6^^-^^13^^]^. It is postulated that exposure of susceptible person to the triggering pathogen may cause release of certain inflammatory mediators which can provoke vasculitis as the main pathological process with resultant clinical manifestations^[^^14^^]^.

 In this study we determined the distribution of human leukocyte B antigens among 90 Iranian patients with diagnosis of KD according to American Heart Association criteria^[^^15^^]^^. ^We used the PCR-SSP technique as described in literature for antigen typing^[^^16^^-^^19^^]^.

## Subjects and Methods

**The study population:** The study population consisted of 90 Iranian patients with final diagnosis of KD at the time of hospital discharge between 1991 and 2008 who were under follow up of pediatric immunology and pediatric cardiology services of Shiraz University of Medical Sciences, Shiraz, Iran. One hundred and nineteen patients were found to fulfill classic criteria of Kawasaki disease according to American Heart Association, among which 90 patients included in our study. Exclusion criteria were all patients presenting less than 4 criteria of Kawasaki. All of the patients were residents of south Iran. Informed consent for participation was obtained from the parents or their guardians. HLA-B distribution of 89 healthy Iranians was used as control^[^^20^^]^. The protocol of the study was approved by Ethical Committee of Shiraz University. 

**Method:** Two samples of one milliliter of blood was obtained from each subject and preserved in -20^⁰^C for Antigen typing by PCR. 

*DNA extraction*: Total DNA was extracted from 200 

μL of whole blood by column based method using amplification DNA blood mini Kit (Qiagnene, England). 

***Determination of DNA concentration:*** DNA concentration was measured by absorbance of the samples at 260 nm and 280 nm versus distilled water as blank. Optical density ratio between 260 nm/280 nm of all samples was above 1.8. The following formula was used to quantify DNA concentration. DNA (μg/ml)=Optical density 260 nm×dilution factor×50. 

 Finally DNA concentration was adjusted to 50-100 μg/ml with distilled water and stored at -20^0^C for analysis.

**Fig. 1:**
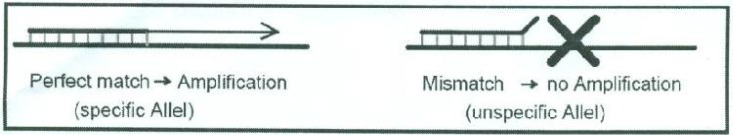
Principle of PCR–SSP

***Antigen-B typing*** **:** Typing was performed by method of PCR-SSP, using BAG HISTO type low resolution kit (BAG Health Care GmbH, Germany). Genomic DNA (50-100 μg/ml) was amplified by PCR–SSP method with 48 pairs of sequence specific primers. Internal positive control was included in each reaction. A total of 48 reactions were done for each sample. This method is based on the fact that primer extension and hence successful PCR relies on an exact match at the 3-end of both primers. So, only if the primers entirely match the target sequence, amplification is obtained (Fig.1). For the PCR procedure the following components were included in 10 μL of reaction mixture: 1 × Polymerase chain reaction buffer (includes dNTps^1^, MgC12), Taq DNA polymerase, internal and sequence specific primers, template DNA. Amplification of PCR-SSP was carried out by using Eppendorf Mastercycler.

**Fig. 2:**
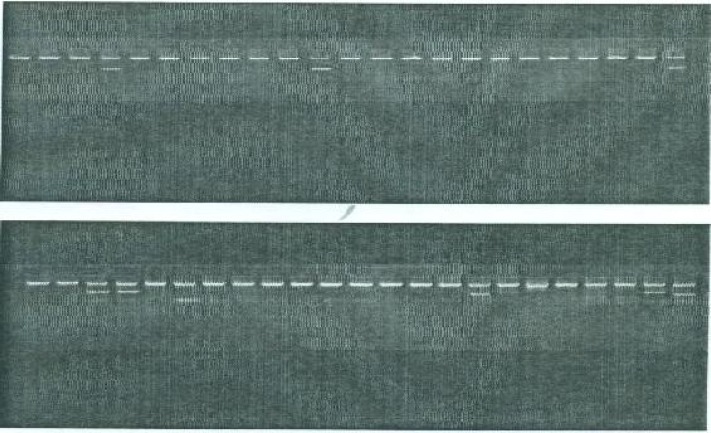
Gel electrophoresis picture of PCR products

***Gel electrophoresis*** **:** PCR products (48 for each sample) were subjected to 3% Agarose gel electrophoresis at 100-150 volt for 30-60 minutes. Then after termination of the run, the gels were stained in ethidium bromide solution for 30 minutes, and under ultraviolet illumination (220-310 nm) PCR products were visualized (Fig. 2).

***Documentation and interpretation:*** For interpretation of the results, specific tables and evaluation diagram were used.

Only bands with correct size were considered as positive reaction. Internal positive control bands were also observed in all reactions. For further reassurance, score evaluation software (BAG Health Care, Germany) was used for analysis of the results. The control group included 89 healthy Iranians who were residents of Shiraz and selected from a population pool on whom a genetic study was done by Farjadian et al in the area^[^^20^^]^. Data were analyzed by Pearson chi-square test and Fisher's exact test according to the frequency specific type antigen in the patients. SPSS version 15 was used for statistical analysis and a *P* value less than 0.05 considered statistically significant. 

## Findings

The frequency of human leukocyte antigen type B of the patients and the control subjects is presented in Table 1. Comparison between frequency of 30 types of B* antigens among patients and control group revealed only 7 (3.9%) patients with positive antigen-B40* type, while 18 (10%) subjects of control group had this antigen with a statistically significant difference (95%CI: 0.13-0.84, RR=1.15 and *P*= 0.02). The frequency of the other 29 types of human leukocyte antigen type B had no statistically significant difference between the patients and control group (Table 2).

## Discussion

While most of the early and late manifestations of KD are controlled with the use of intravenous immunoglobulin and acetylsalicylic acid, the etiology and possible predisposing factors are still undetermined. For development of KD, different etiological factors such as bacteria, viruses, mycoplasma, superantigens or even rug shampoo have been accused^[^^1^^]^.

**Table 1:** Prevalence of complete Kawasaki criteria in the patients

**Parameter**	**Frequency**
**Sex: male/female **	1:4
**Lymph node **	74%
**Conjunctivitis **	94%
**Oral mucosa involvement**	90%
**Extremity involvement **	74%
**Rash **	78%
**Cardiac involvement including coronary artery aneurism**	26%
**Coronary artery aneurism **	0.06%

**Table 2:** Prevalence and percentage of human leukocyte antigen type B in the patients and controls

**Human Leukocyte Agntigen -B**	**Patients (90)** **n**	**%**	**Controls (89)** **n**	**%**	***P*** ** value**
*07	9	5	4	2.2	0.2
*08	5	2.8	11	6.2	0.1
*13	6	3.3	2	1.1	0.3
*14	4	2.2	4	2.2	1.0
*15	3	1.7	3	1.7	1.0
*18	9	5	16	9	0.1
*27	2	1.1	4	2.2	0.4
*35	43	23.9	35	19.7	0.3
*.38	4	2.2	-	0	0.1
*39	3	1.7	1	0.6	0.6
*40	7	3.9	18	10.1	0.02
*41	7	3.9	1	0.6	0.07
*42	-	0	2	1.1	0.2
*44	8	4.4	5	2.8	0.4
*45	1	0.6	3	1.7	0.4
*49	2	1.1	1	0.6	1.0
*50	7	3.9	2	1.1	0.2
*51	17	9.4	21	11.8	0.5
*52	8	4.4	7	3.9	1.0
*53	5	2.8	13	7.3	0.05
*55	6	3.3	7	3.9	0.8
*56	1	0.6	1	0.6	1.0
*57	4	2.2	3	1.7	1.0
*58	7	3.9	9	5.1	0.6
*73	3	1.7	1	0.6	0.3
*81	-	0	4	2.2	0.06
*37	5	2.7	0	0	0.06
*54	2	1.1	0	0	0.5
*36	1	0.6	0	0	1.0
*47	1	0.6	0	0	1.0

Rare presentation of KD in infants younger than 4 months may suggest protective role of maternal antibodies^[^^14^^]^. Having higher incidence among those with Asian background, especially 5000-6000 new cases annually in Japan, and the greatest occurrence in twins, suggest the role of a genetic predisposition^[^^10^^]^. This genetic background may predispose the patient to a super-antigen driven response with a selective expression of immune system and then the release of certain mediators of inflammation. The production of various inflammatory cytokines and elevated level of matrix metalloproteinases may mediate the vascular endothelial damages^[^^14^^,^^21^^]^. Accordingly, the presence of certain human leukocyte antigens may increase susceptibility of the patients to KD or may protect them against the disease or its complications. Kato et al demonstrated increased presence of human leukocyte antigen BW22 in patients with KD in comparison with control group (*P*<0.0005)^[^^6^^]^. Krensky et al in their report from Boston hospital showed increased prevalence of BW51 antigen among Kawasaki patients (*P*<0.002)^[^^7^^]^. While Kaslow et al in their study reported that the combination of different human leukocyte antigens may have a role. Chang et al from China, Hong Kong, did not show any association between presence of human leukocyte antigens, including type B and KD^[^^8^^-^^9^^]^.

 Recently Maggioli et al reported the possible role of HLA class III region and susceptibility to KD^[^^22^^]^.

 Also with respect to development of coronary artery aneurysms, polymorphism of transmembrane region of human leukocyte antigen genes and presence of certain alleles with increased susceptibility or a possible protective role for coronary complication in KD has been suggested^[^^23^^-^^25^^]^. So far studies from around the world have shown varied and inconsistent association between KD and single or summation of alleles (halotype)^[^^26^^]^. However, the possibility of a protective role for human leukocyte antigen in KD has not been reported as far as we are aware. For certain infectious diseases Cresio Alves et al have reviewed the association between human leukocyte antigen genes and increased susceptibility or protection against certain infections and their complication^[^^27^^]^. In their report, among Chilean patients with Chagas disease the incidence of cardiomyopathy was lower in those patients with antigen type B40*, however a report from Venezulea did not confirm this finding. Also for leprosy a protective role for type-A and type-B summations (halotype) has been suggested. The prevalence of antigen-B40* among our control group was about 10.1%. This antigen also has been the most frequent allele among Mexican, Korean, Chinese from Hainan province and Omanis^[^^9^^,^^12^^,^^28^^,^^29^^]^. Considering the higher frequency of antigen type-B40^+^ allele among our control subjects, may assume the possibility of a protective role for this antigen in our area. However, more studies are needed to find out the role of B-40* antigen and KD among mentioned ethnic groups with higher frequency of this antigen. 

Limitation of study: 

 It is shown that many genetic characters can be transferred in halotype pattern, i.e. summation of multiple genes. The major limitation of our study is that we checked only the type-B antigens. Certainly further studies are needed t*o *determine the possibility of a protective role for certain single gene or summation of genes for KD in our area.

## Conclusion

The presence of higher frequency of allele type-B40* in the control group may represent a protective role for this antigen with resultant decreased susceptibility to KD in our area. Presence or lack of certain human leukocyte antigens has been assumed for expression of certain genes which can induce production of mediators of inflammatory response in this disease.
